# Comparison of different transcatheter interventions for treatment of mitral regurgitation: A protocol for a network meta-analysis

**DOI:** 10.1097/MD.0000000000023623

**Published:** 2020-12-11

**Authors:** Bowen Zhang, Muyang Li, Yingying Kang, Lina Xing, Yu Zhang

**Affiliations:** a Department of Cardiothoracic Surgery, Wuwei People's Hospital, Gansu; b The Second Clinical Medical College of Lanzhou University; c School of Basic Medical Sciences, Lanzhou University; d Department of Thoracic Surgery, First Hospital of Lanzhou University, Lanzhou, China.

**Keywords:** efficacy, mitral regurgitation, network meta-analysis, safety, transcatheter mitral valve therapy

## Abstract

**Background:**

The arrival of transcatheter mitral valve therapies has provided feasible and safe alternatives to medical and surgical treatments for mitral regurgitation. The aim of this study is to estimate the relative efficacy and safety of different transcatheter mitral valve therapies for mitral regurgitation patients through network meta-analysis.

**Methods:**

A systematic search will be performed using PubMed, EMBASE, the Cochrane Library, Web of Science, Chinese Biomedical Literature Database, and China National Knowledge Infrastructure to include random controlled trials and nonrandom controlled trials comparing the efficacy and safety of different transcatheter mitral valve techniques. The risk of bias for the included nonrandom controlled studies will be evaluated according to Risk of Bias in Non-randomized Studies - of Interventions. For random controlled trials, we will use Cochrane Handbook version 5.1.0 as the risk of bias tool. A Bayesian network meta-analysis will be conducted using R-4.0.3 software. Grading of recommendations assessment, development, and evaluation will be used to assess the quality of evidence.

**Results:**

The results of this network meta-analysis will be submitted to a peer-reviewed journal for publication.

**Conclusion:**

This study will provide broad evidence of efficacy and safety of different transcatheter mitral valve therapies for treatment of mitral regurgitation and provide suggestions for clinical practice and future research.

**Protocol registration number:**

INPLASY2020110034.

## 1 Introduction

Mitral regurgitation (MR) is one of the most common valvular heart diseases with retrograde blood flow from the left ventricle into the left atrium in ventricular systole.^[1,2]^ It is characterized as primary or secondary according to the cause of disease.^[3]^ Primary MR with the pathology of the mitral valve (MV) structure itself while secondary MR is caused by distortion of the apparatus and/or function of the left ventricle.^[4,5]^ Untreated, severe MR results in high mortality and frequent hospitalization for treatment of heart failure.^[6]^ Yearly mortality rates of patients aged 50 years or older with medical treatment are about 3% for moderate MR and about 6% for severe MR.^[1]^ Surgery is still the first-line treatment to improve symptoms and prevent heart failure; however, a high percentage of patients with MR are not suitable for open-heart surgery due to high operative risk, mainly related to advanced age, impaired left ventricular function, and complications,^[7]^ which posed an important therapeutic challenge. In recent years, the arrival of transcatheter MV therapies has provided feasible and safe alternatives to medical and surgical treatments, which take advantage of the less invasive approaches such as transesophageal echocardiography and fluoroscopy for monitoring the procedural steps to maximize the outcomes and minimize the complications.^[8,9]^ Transcatheter techniques to treat MR are based on the existing surgical heart valve procedures including leaflet and chordae repair, annuloplasty, left ventricular remodeling and replacement, and there were several corresponding devices which combines the respective advantages of cardiac surgery have aroused great interest.^[10–12]^

There was lacking of head-to-head comparisons between different transcatheter techniques and paired meta-analysis has the disadvantage of not being able to simultaneously integrate all types of transcatheter methods from different original studies.^[13,14]^ Network meta-analysis (NMA) has become gradually popular to estimate healthcare interventions since it allows to assess the relative effectiveness among all interventions and rank ordering of the interventions in the absence of direct evidence, which will play an increasingly supreme role in clinical decision-making because many indications have multiple therapeutic options that were lacking of comparisons with each other.^[15–17]^ Even when the results of the direct comparisons are conclusive, combining them with indirect evaluations in a mixed treatment comparison may yield more refined evaluations.^[18]^

The aim of this study is to estimate the relative efficacy and safety of different transcatheter MV therapies for MR patients through NMA in a Bayesian mixed-treatment framework.

## 2 Methods

### 2.1 Registration and ethical approval

This NMA protocol extension statement is according to the preferred reporting items for systematic review and meta-analysis protocols.^[19]^ We registered our protocol on the INPLASY website, and the registration number is INPLASY2020110034. The ethical approval and patient informed consent are unnecessary because this study is based on published studies.

### 2.2 Date source

We will perform a systematic search via PubMed, EMBASE, the Cochrane Library, Web of Science, Chinese Biomedical Literature Database, and China National Knowledge Infrastructure. Besides, the reference lists of included studies and other relevant articles will be retrieved for supplement.

### 2.3 Search strategy

Search terms will be: mitral regurgitation, transcatheter, percutaneous, mitral valve repair, chordae tendineae implantation, annuloplasty, and mitral valve replacement.

Full details of the search strategy with respect to PubMed was as follows:

#1 “Mitral Valve Insufficiency”[Mesh]

#2 Mitral Valve Regurgitation[Title/Abstract] OR Regurgitation, Mitral Valve[Title/Abstract] OR Valve Regurgitation, Mitral[Title/Abstract] OR Mitral Valve Insufficiency[Title/Abstract] OR Insufficiency, Mitral Valve[Title/Abstract] OR Valve Insufficiency, Mitral[Title/Abstract] OR Mitral Regurgitation[Title/Abstract] OR Regurgitation, Mitral[Title/Abstract] OR Mitral Valve Incompetence[Title/Abstract] OR Incompetence, Mitral Valve[Title/Abstract] OR Valve Incompetence, Mitral[Title/Abstract] OR Mitral Incompetence[Title/Abstract] OR Incompetence, Mitral[Title/Abstract] OR Mitral Insufficiency[Title/Abstract] OR Insufficiency, Mitral[Title/Abstract]

#3 #1 OR #2

#4 Percutaneous edge-to-edge mitral valve repair [Title/Abstract] OR MitraClip[Title/Abstract] OR Pascal[Title/Abstract] OR ValveClamp[Title/Abstract]

#5 Transcatheter chordae tendineae implantation[Title/Abstract] OR NeoChord∗[Title/Abstract] OR Harppon[Title/Abstract]

#6 Transcatheter mitral annuloplasty [Title/Abstract] OR Carillon[Title/Abstract] OR Mitralign[Title/Abstract] OR Cardioband[Title/Abstract] OR ARTO system[Title/Abstract]

#7 Transcatheter mitral valve replacement[Title/Abstract] OR Transcatheter valve in valve[Title/Abstract] OR Transcatheter valve in ring[Title/Abstract] OR Tendyne[Title/Abstract] OR Intrepid[Title/Abstract]

#8 #4 OR #5 OR #6 OR #7

#9 #3 AND #8

### 2.4 Eligibility criteria

#### 2.4.1 Type of studies

Randomized and nonrandomized controlled studies will be both included, and related systematic reviews or meta-analysis will be also included for retrieving their applicable reference.

#### 2.4.2 Type of participants

Patients with MR confirmed by clinical or transesophageal echocardiography.

#### 2.4.3 Type of interventions

We will include studies that used at least one of the interventions about transcatheter MV technologies, as follows:

1. Percutaneous edge-to-edge mitral valve repair, which may use device names to represent this procedure including Mitraclip, Pascal, or ValveClamp;
2. Transcatheter chordae tendineae implantation, the device including NeoChord, Neochordae, or Harppon;
3. Transcatheter mitral annuloplasty, which may use device names to indicate this procedure including Carillon, Mitralign, Cardioband, or ARTO system.
4. Transcatheter Mitral Valve Replacement, transcatheter valve in valve, transcatheter valve in ring, the device including Tendyne or Intrepid.

#### 2.4.4 Type of outcomes

The primary outcomes include all-cause mortality, unplanned rehospitalisation for cardiovascular reasons, and mitral valve reintervention. The secondary outcomes include rate of periprocedural adverse events and serious adverse device effects, change in NYHA class, quality of life, biological parameters like B-type natriuretic peptide, and additional secondary outcomes include the situation of left and right cardiac chamber remodeling and restoration of function, and change in 6 minute walk test.^[20,21]^ All of the follow-up time are comparable.

#### 2.4.5 Other criteria

We will include studies with language of English or Chinese and there will be no restrictions on the year of publication and publication status.

### 2.5 Study selections

The identified records will be imported into EndNote X9 (Thomson Reuters (Scientific) LLC Philadelphia, PA) software for management. The first need to do is to remove the duplicate records and then there will be 2 independent authors who select the potential studies by screening titles and abstracts. The records that do not meet the inclusion criteria will be excluded. After that the full text of each potential study will be assessed by the same 2 investigators to judge whether they meet the eligibility criteria. The disagreements between 2 authors will be resolved by discussion with a third reviewer.

### 2.6 Data items

We will use Microsoft Excel 2019 (Microsoft, Redmond, WA, www.microsoft.com) to create a standard data extraction form and collect the required data. Two independent authors will extract the following data including first author, year of publication, country of corresponding author, number of authors, journal of publication, funding, location, study design, study period, study arms, sample, mean age, gender, MR characteristics, methods of intervention and comparison, device used, median follow-up and outcomes, and the discordance will be resolved by discussion with a third author.

### 2.7 Risk of bias of individual studies

The risk of bias of included nonrandomized studies will be assessed according to the tool named Risk Of Bias in Non-randomized Studies - of Interventions (ROBINS-I), which is divided into 7 domains including bias due to confounding (preintervention), bias in selection of participants into the study (preintervention), bias in classification of interventions (at intervention), bias due to deviations from intended interventions (postintervention), bias due to missing data (postintervention), bias in measurement of outcomes (postintervention), bias in selection of the reported result (postintervention), and finally with an assessment of overall risk of bias.^[22]^ The risk of bias will be evaluated as low, moderate, serious, critical risk of bias, and no information.

The risk of bias of included randomized studies will be evaluated using the tool from Cochrane Handbook version 5.1.0 in 6 domains, including method of random sequence generation (selection bias), allocation concealment (selection bias), blinding (performance bias and detection bias), incomplete outcome data (detection bias), selective reporting (detection bias), and other source of bias.^[23]^ We will assess risk of bias as low, high, or unclear risk of bias.

The risk of bias assessment will be completed by 2 independent reviewers, and disagreements will be resolved by a third reviewer.

### 2.8 Geometry of the network

A network plot will be conducted to describe and indicate the geometry of different transcatheter MV therapies using STATA V.15.0 (Stata Corporation, College Station, TX, USA Stata) software. Nodes will be used to stand for different transcatheter interventions and edges used to represent the head-to-head comparisons between interventions. The size of nodes and thickness of edges severally represent the sample sizes of intervention and numbers of included trials.

### 2.9 Statistical analysis

#### 2.9.1 Pairwise meta-analysis

We calculated the average odds ratio with the 95% confidence interval (CI) for dichotomous outcomes and calculated the average standard mean difference (or the weighted mean difference which all studies used the same scale) with 95% CI for continuous outcomes. The heterogeneity within each pairwise comparison will be evaluated by I^2^ statistics. If I^2^ ≤50%, it suggests that there is negligible statistical heterogeneity and the fixed effects model will be used for meta-analysis. If I^2^ > 50%, it indicates that there is possible statistical heterogeneity existed and we will explore the sources of heterogeneity by subgroup analysis and meta-regression using effect modifiers. If there is no clinical heterogeneity, the random effects model will be used to perform meta-analysis.

#### 2.9.2 Network meta-analysis

We will perform a Bayesian NMA using package “*gemtc*” version 0.8 to 7 of R-4.0.3 software (R Foundation for Statistical Computing, Vienna, Austria).^[24]^ The Markov chains Monte Carlo sampler will be used to generate samples via the function *mtc.run.* Four Markov chains will be run concurrently. We will set 5000 simulations for each chain as the “burn-in” period. Then 50,000 subsequent simulations will be used as a base in posterior summaries. Brooks-Gelman-Rubin plots method will be used to evaluate the model convergence. And the inconsistency between direct and indirect comparisons will be assessed by a node-splitting method if there is a loop connecting 3 arms.

Rank probabilities will be calculated to present the probability for each treatment to be the best, second best, and so on. The recommendation of clinical decisions with respect to the choice of treatments can be based on the results of rank probabilities when different treatments have small differences in effect size.^[25]^ A matrix of the treatment rank probabilities and a plot of the rank probabilities can be provided by the “*gemtc*” package.

#### 2.9.3 Sensitivity and subgroup analyses

We need to solve heterogeneity because it is only when the included studies have the least heterogeneity, the credibility of the synthesized effect size is high, and sensitivity and subgroup analyses are the most common approaches used to solve heterogeneity. If the results of NMA are positive and the number of included studies is over 3, we will analyze the sensitivity using STATA V.15.0 software. The sensitivity analysis is performed by excluding study one by one. The sensitivity is low and the results are of stability and reliability if there are no significant changes that appear in the results before and after the exclusion; if not, it indicates a high sensitivity and unstable result.^[26]^ And in this study, year of publication, country of corresponding author, type of study design, mean age, and length of follow-up time will be considered and designed for subgroup analysis to find the possible sources significant heterogeneity.

#### 2.9.4 Funnel plot analysis

Begg and Egger funnel plot method will be performed to help distinguish asymmetry caused by publication bias.^[27,28]^ And whether there will be a small effect between intervention networks will be identified by the comparison-adjusted funnel plot.

### 2.10 Quality of evidence

We will use the grading of recommendations assessment, development, and evaluation (GRADE) approach to evaluate the quality of the evidence. The GRADE approach has considerations of 5 aspects, including study limitations, consistency of effect, imprecision, indirectness, and publication bias, for the evaluation of the quality of the body of evidence about each outcome.^[29]^ It is categorized as 4 levels: high level, moderate level, low level, and very low level.

## 3 Discussion

Although the gold standard for MR remains surgical intervention with mitral valve repair/replacement, there is an important therapeutic challenge with the growing number of patients who suffer from symptomatic severe MR while with contraindications to surgery or high operative risk pose.^[8,30]^ The arrival of transcatheter MV repair or replacement for MR through small arterial and venous entry sites avoids risks associated with open heart surgery.^[31]^ It has been confirmed in RCT that transcatheter MV repair in prohibitive surgical risk patients linked with safety and good clinical outcomes, including reductions in rehospitalization, improvements in function, and favorable ventricular remodeling, at 1 year.^[32]^ Transcatheter MV repair using a percutaneous edge-to-edge technique is the most widely available choice at the moment, while other transcatheter MV repair methods such as annuloplasty and chordal implantation are watchable alternatives. Besides, emerging technologies in transcatheter MV replacement are speedily establishing their roles in the field of MR therapy.^[12,30]^ For all we have known, this study will first compare the efficacy and safety of different transcatheter MV therapies for treatment of MR using Bayesian NMA.

This NMA will summarize the direct and indirect evidence to evaluate and compare the efficacy and safety of different transcatheter MV techniques. Furthermore, we will evaluate the risk of bias of each included study via ROBINS-I or Cochrane Handbook Version 5.1.0 and assess the quality of evidence using the GRADE framework. We hope that our study will provide suggestions for clinical practice and future research.

## Acknowledgments

The authors thank all investigators and supporters involved in this study.

## Author contributions

BZ and ML planned and designed the study, YK and LX tested the feasibility of the study. YZ provided methodological advice, considered for overall structure of the article, and revised the manuscript. BZ and ML wrote the manuscript. All authors approved the final version of the manuscript.

**Data curation:** Yingying Kang, Lina Xing.

**Investigation:** Bowen Zhang.

**Methodology:** Yu Zhang.

**Resources:** Muyang Li.

**Supervision:** Yu Zhang.

**Writing – review & editing:** Bowen Zhang, Muyang Li.

## References

[1] Enriquez-Sarano M Akins CW Vahanian A Mitral regurgitation. Lancet 2009;373:1382–94. 19356795 10.1016/S0140-6736(09)60692-9

[2] El-Tallawi KC Messika-Zeitoun D Zoghbi WA Assessment of the severity of native mitral valve regurgitation. Prog Cardiovasc Dis 2017;60:322–33. 29174559 10.1016/j.pcad.2017.11.005

[3] Argulian E Borer JS Messerli FH Misconceptions and facts about mitral regurgitation. Am J Med 2016;129:919–23. 27059381 10.1016/j.amjmed.2016.03.010

[4] Gillam LD Marcoff L Hemodynamics in primary mitral regurgitation: support for and challenges to the conventional wisdom. Circ Cardiovasc Imaging 2018;11:e007471. 29449413 10.1161/CIRCIMAGING.118.007471

[5] O’Gara PT Mack MJ Secondary mitral regurgitation. N Engl J Med 2020;383:1458–67. 33027570 10.1056/NEJMcp1903331

[6] Sannino A Grayburn PA Mitral regurgitation in patients with severe aortic stenosis: diagnosis and management. Heart 2018;104:16–22. 28903993 10.1136/heartjnl-2017-311552

[7] Praz F Brugger N Kassar M Interventional treatment of mitral valve regurgitation: an alternative to surgery? Swiss Med Wkly 2019;149:w20023. 30852836 10.4414/smw.2019.20023

[8] Bax JJ Debonnaire P Lancellotti P Transcatheter interventions for mitral regurgitation: multimodality imaging for patient selection and procedural guidance. JACC Cardiovasc Imaging 2019;12:2029–48. 31601378 10.1016/j.jcmg.2019.03.036

[9] Feldman T Leon MB Prospects for percutaneous valve therapies. Circulation 2007;116:2866–77. 18071090 10.1161/CIRCULATIONAHA.106.621375

[10] Bozkurt S Preston-Maher GL Torii R Design, analysis and testing of a novel mitral valve for transcatheter implantation. Ann Biomed Eng 2017;45:1852–64. 28374279 10.1007/s10439-017-1828-2 PMC5527080

[11] Ramlawi B Gammie JS Mitral valve surgery: current minimally invasive and transcatheter options. Methodist Debakey Cardiovasc J 2016;12:20–6. 27127558 10.14797/mdcj-12-1-20 PMC4847963

[12] Herrmann HC Maisano F Transcatheter therapy of mitral regurgitation. Circulation 2014;130:1712–22. 25366833 10.1161/CIRCULATIONAHA.114.009881

[13] Salanti G Indirect and mixed-treatment comparison, network, or multiple-treatments meta-analysis: many names, many benefits, many concerns for the next generation evidence synthesis tool. Res Synth Methods 2012;3:80–97. 26062083 10.1002/jrsm.1037

[14] Rouse B Chaimani A Li T Network meta-analysis: an introduction for clinicians. Intern Emerg Med 2017;12:103–11. 27913917 10.1007/s11739-016-1583-7 PMC5247317

[15] Xu Y Amiche MA Tadrous M Network meta-analysis: an introduction for pharmacists. Int J Clin Pharm 2018;40:942–7. 29785685 10.1007/s11096-018-0656-2

[16] Bafeta A Trinquart L Seror R Reporting of results from network meta-analyses: methodological systematic review. BMJ 2014;348:g1741. 24618053 10.1136/bmj.g1741 PMC3949412

[17] Lu G Ades AE Combination of direct and indirect evidence in mixed treatment comparisons. Stat Med 2004;23:3105–24. 15449338 10.1002/sim.1875

[18] Li L Tian J Tian H Network meta-analyses could be improved by searching more sources and by involving a librarian. J Clin Epidemiol 2014;67:1001–7. 24841794 10.1016/j.jclinepi.2014.04.003

[19] Shamseer L Moher D Clarke M Preferred reporting items for systematic review and meta-analysis protocols (PRISMA-P) 2015: elaboration and explanation. BMJ 2015;350:g7647. 25555855 10.1136/bmj.g7647

[20] Piriou N Al Habash O Donal E The MITRA-HR study: design and rationale of a randomised study of MitraClip transcatheter mitral valve repair in patients with severe primary mitral regurgitation eligible for high-risk surgery. EuroIntervention 2019;15:e329–35. 30987963 10.4244/EIJ-D-18-01086

[21] Kheiri B Zayed Y Barbarawi M Interventions for secondary mitral regurgitation in patients with heart failure: a network meta-analysis of randomized controlled comparisons of surgery, medical therapy and transcatheter intervention. Cardiovasc Revasc Med 2020;21:155–63. 31201060 10.1016/j.carrev.2019.04.008

[22] Sterne JA Hernán MA Reeves BC ROBINS-I: a tool for assessing risk of bias in non-randomised studies of interventions. BMJ 2016;355:i4919. 27733354 10.1136/bmj.i4919 PMC5062054

[23] Higgins J Cochrane Handbook for Systematic Reviews of Interventions Version 5.0.1. The Cochrane Collaboration Naunyn-Schmiedebergs Archiv für experimentelle Pathologie und Pharmakologie 2008;5:S38.

[24] Shim SR Kim SJ Lee J Network meta-analysis: application and practice using R software. Epidemiol Health 2019;41:e2019013. 30999733 10.4178/epih.e2019013 PMC6635665

[25] Rubin GDB Inference from iterative simulation using multiple sequences. Statist Sci 1992;7:457–72.

[26] Li L Guo LY Mao J A network meta-analysis protocol of adjuvant chemotherapy for unresectable patients with advanced gastric cancer. Medicine (Baltimore) 2019;98:e16108. 31305394 10.1097/MD.0000000000016108 PMC6641784

[27] Begg CB Mazumdar M Operating characteristics of a rank correlation test for publication bias. Biometrics 1994;50:1088–101. 7786990

[28] Egger M Davey Smith G Schneider M Bias in meta-analysis detected by a simple, graphical test. BMJ 1997;315:629–34. 9310563 10.1136/bmj.315.7109.629 PMC2127453

[29] Puhan MA Schünemann HJ Murad MH A GRADE Working Group approach for rating the quality of treatment effect estimates from network meta-analysis. BMJ 2014;349:g5630. 25252733 10.1136/bmj.g5630

[30] Sidhu RS Tyrrell BD Welsh RC Transcatheter mitral valve intervention for chronic mitral regurgitation: a plethora of different technologies. Can J Cardiol 2018;34:1200–9. 30170675 10.1016/j.cjca.2018.04.034

[31] Markham R Kyranis S Aroney N Transcatheter mitral valve intervention: an emerging treatment for mitral regurgitation. Intern Med J 2018;48:382–90. 29623986 10.1111/imj.13750

[32] Lim DS Reynolds MR Feldman T Improved functional status and quality of life in prohibitive surgical risk patients with degenerative mitral regurgitation after transcatheter mitral valve repair. J Am Coll Cardiol 2014;64:182–92. 24184254 10.1016/j.jacc.2013.10.021

